# What Drives Consumer Purchasing Intention in Live Streaming E-Commerce?

**DOI:** 10.3389/fpsyg.2022.938726

**Published:** 2022-06-27

**Authors:** Chenglin Qing, Shanyue Jin

**Affiliations:** ^1^Department of Business Administration, Honam University, Gwangju, South Korea; ^2^College of Business, Gachon University, Seongnam, South Korea

**Keywords:** live streaming e-commerce, purchasing intention, perceived ease of use, perceived trust, perceived usefulness, perceived value, quality

## Abstract

The live streaming e-commerce market continues to grow with the rapid increase in contactless communication due to COVID-19. Live streaming e-commerce goes beyond the confines of traditional e-commerce of simply selling goods or services. It supplies information and allows synchronous information exchange between the online viewer (consumer) and the Internet celebrity, who influences the consumers information behavior and ultimately contributes to the long-term profit generation of the company. From online commerce to new retail and live streaming, China has been at the forefront of innovation in online commerce worldwide. Therefore, this study focuses on investigating the influences of live streaming e-commerce quality, such as service quality (SEQ), information quality (IQ), and system quality (SQ), on consumer purchase intention in China by applying the extended model of technology acceptance. Furthermore, this study analyzes the paths through which the live streaming e-commerce quality affects the consumer purchase intention, based on paths of perceived usefulness (PU), perceived ease of use (PEU), perceived trust (PT), and perceived value (PV). To analyze this study, the data were collected from 231 customers who had used live streaming e-commerce apps in China (e.g., TikTok, Taobao, and Jingdong) and empirical analysis was applied. The results were summarized as follows: SQ is positively related to PU and PT. Similarly, IQ is positively related to PEU and PT. In addition, SEQ is also positively related to PU and PEU. Moreover, SQ affected the PV through PU and PT significantly and ultimately influenced consumer PI. Also, the path in which SEQ affects PU, which in turn affects PV, and ultimately PI, was found. This study found out the paths of how the live streaming E-commerce quality affects the consumer purchasing intention and contributes to the research field of e-commerce and consumer behaviors. Additionally, the results of this study give useful marketing suggestions for relevant financial institutions and enterprises.

## Introduction

The ongoing COVID-19 pandemic has affected most parts of the world, posing unprecedented economic challenges. Simultaneously, emerging business forms, such as residential and non-contact economies, have changed consumption and working patterns. As an emerging consumption mode, live streaming e-commerce is gaining traction. This form of e-commerce furthers innovative online business. From e-business to new retail and live streaming, China has been at the forefront of innovation in online business worldwide. Live streaming e-commerce is an upgraded form of social commerce, given that it includes a more interactive form of content. It has three primary advantages: shortening the demand chain from researching products to making purchase decisions, condensing the supply chain by eliminating intermediaries, and realizing sales in a scenario-based manner (Hu and Chaudhry, 2020). With further maturity of technologies, such as fifth-generation mobile communication technology, virtual reality, and augmented reality, live streaming can be more interactive and richer in content presentation. This will likely be the norm for e-commerce in the future.

Live streaming is a part of online commerce and marketing sales. It involves instant orders, live product demonstrations, real-time question and answer, limited-time price promotions, real-time communication, and online streaming services hosted by online stores (Wongkitrungrueng and Assarut, 2020). Live streaming eliminates the communication barrier between brands and consumers and focuses on promoting short-term sales by combining brand marketing and performance marketing to enhance corporate marketing efficiency. Specifically, companies combine brand marketing and performance marketing to deliver comprehensive brand stories to consumers, introduce emotional and cultural elements to products to receive immediate feedback, and create a ripple effect on new products through product demonstrations, ultimately increasing marketing efficiency in China (Chung and Wei, 2020). Through various types of live streams, market segmentation and positioning can be adjusted to specific needs, and brand loyalty can be derived by establishing individual features for consumers.

Compared with the first quarter of 2020, Chinese online short video e-commerce and live streaming users increased by more than 4.9% in the second quarter. Meanwhile, the number of people using live streaming e-commerce totaled 309 million, up by 16.7% from the first quarter. Moreover, the number of people using online commerce totaled 749 million, accounting for 79.7% of all Internet users (Grand View Research, 2020). Therefore, live streaming e-commerce was the fastest-growing trend in 2020. In the world market, China has ranked first for seven consecutive years and is estimated to provide important support for new development. Live streaming e-commerce is rapidly developing, with the penetration rate of related apps steadily increasing, and the form and characteristics of products diversifying. With the strengthening of online communication, the number of live streaming users has been steadily increasing. This suggests that live streaming can serve as a window to provide information on products/services, exchange emotions and opinions, and manage corporate brands, such as through social networking services, and go beyond a channel that just sells products (Doha et al., 2019).

Although research is being actively conducted in line with the market growth, and despite an increasing interest in live streaming e-commerce, research remains limited. Most of the research related to live streaming e-commerce has been based on consumer decision-making models (Kumar et al., 2018; Pousttchi and Dehnert, 2018). The consumer value of live streaming e-commerce suggests that it provides the value (intrinsic) that enables the information of the product to be found quickly and efficiently and that it also provides interesting value (external) by focusing on something by itself through online searches (Ram and Xu, 2019).

This study investigates the impact of live streaming e-commerce quality, such as service quality (SEQ), information quality (IQ), and system quality (SQ), on consumer purchase intention (PI) in China by applying the extended technology acceptance model (ETAM). Furthermore, this study analyzes the paths through which the live streaming e-commerce quality affects the consumer purchase intention. We found differences in the effect of e-commerce live streaming quality on PI, based on the paths of perceived value (PV), perceived ease of use (PEU), perceived trust (PT), and perceived usefulness (PU).

This study found out the paths of how the live streaming e-commerce quality affects the consumer purchasing intention and contributes to the research field of online commerce and consumer behaviors. Additionally, the results provide useful marketing suggestions for relevant financial institutions and enterprises.

## Theoretical Background and Hypotheses

### Extended Technology Acceptance Model

Davis et al. (1989) confirmed that PEU in the early technology acceptance model (TAM) directly affects behavioral intention and PU, and they consequently proposed a TAM that omitted the attitude variable. PU refers to a user’s expectation of how much new technology helps and improves performance (Davis, 1989; Shih, 2004). It is a strong factor affecting the acceptance of new technology, and its effect as a model with a high predictive power of purchase and use intentions for innovative products or services has been verified (Yadav and Mahara, 2019). PEU is defined as the extent to which individuals believe that they are free from physical and mental endeavors while using a particular system (Venkatesh and Davis, 1996). In a follow-up study, Venkatesh and Davis (2000) presented an ETAM with external variables to compensate for the limitations of the existing TAM. The expansion of TAM can be categorized into studies that propose new antecedent variables (Van der Heijden, 2004; Petter et al., 2008; Venkatesh et al., 2012) and apply new parameters, in addition to the PU and PEU (DeLone and McLean, 2003; Sánchez and Hueros, 2010; Venkatesh et al., 2012).

There have been numerous studies on the attitudes and behaviors of consumers using new products or services in both the field of social sciences and business administration. In particular, these studies have been on consumers’ acceptance and intention to use newly launched products and services, applying innovative technologies. Such studies were initially focused on antecedent factors affecting consumer attitudes and behavioral intention and were later extended to the study of the information technology acceptance process. In addition, several theories have been presented to explain that users accept new technologies and their intention to use them. A well-known theory is the TAM, which has been academically acknowledged for its excellence as a model with high explanatory power on how users accept and use new technologies (Petter et al., 2008). TAM is one of the information systems theories that can be used to understand and predict customers’ behavior based on the theory of reasoned action (TRA), and TAM suggests that both PU and PEU impact the usage of new technologies and acceptance (Venkatesh and Davis, 1996). PU and PEU affect consumers’ attitudes in accepting innovative technologies, which in turn affect their intention to use, and are considered to be important factors affecting consumers’ decisions to use or reject new products and services.

Perceived usefulness refers to the degree of an individual’s belief that using new information technology system will increase personal work performance. The users’ acceptance of the new information technology system is negatively affected by a low PU. A high PU indicates that a new product or service will enhance convenience or value. Therefore, PU is a key factor that explains user acceptance of new technologies, especially in the new online shopping environment. Davis et al. (1989) verified in their study that both PEU and PU are key variables that impact the intention to use and argued that TAM is the most powerful model, which can explain and predict customer’s acceptance of new technologies. PEU is the degree of individual’s belief that using new information technology system leads to being free of effort or difficulty. If the user feels that using a specific new information technology system in a shopping environment is easier, the intention to use increases, and there is a high probability that it will be used.

Venkatesh and Davis (2000) proposed an ETAM by adding various external variables to the existing TAM. The attitude factors were removed. and instead, external variables involving social influence and cognitive instrumental processes were added to verify their effect on PU. Adding experience and voluntariness, the roles of external variables on the intention of using information technologies were also verified. Recently, various studies on products and services that are applications of new innovative technologies are pursued by expanding and verifying external variables based on the integrated model with TAM. In particular, various studies on services offered by several platforms are conducted with the improved reliability of commercial mobile technologies to verify the effect on attitudes and behavioral intentions. In studies applying the ETAM (Venkatesh and Davis, 2000), variables such as SQ, IQ, SEQ, and outcome quality are used as system characteristic factors, and social characteristic factors such as subjective norms, as well as personal characteristic factors, such as innovativeness, motivation, and self-efficacy, were also used (Fayad and Paper, 2015). Furthermore, various studies are under way to verify the mediating effects of belief variables, such as trust, pleasure, value, and risk, on user acceptance.

In the e-commerce of live streaming environment, trust is an essential factor in determining consumers’ expectations and consumer behavior, since it is generally viewed as a belief in the transaction with the seller, as well as a belief in the transaction platform applying the innovative technologies. Ozdemir and Sonmezay (2020) stated that trust acts as a factor that reduces perceptions of risk, according to the common acceptance of the premise that trust presupposes a situation related to risk. In the live streaming e-commerce environment based on innovative technologies, trust is an essential factor, which is being evaluated more due to the uncertainties that are characteristic of these services (Lăzăroiu et al., 2020). Therefore, trust plays a decisive role in consumer buying behavior and PI. In the current business environment where a lot of information is on offer, consumers make purchase decisions centered on the reliability of the information on products and services. In live streaming e-commerce, in particular, trust in information providers is deemed very important because users are unable to see or directly test the products. In the process of accurately identifying and delivering the source and format of information provided online, trust plays a major role in helping consumers reduce perceived risks.

In the existing research, the factors are classified according to the characteristics of the features affecting the TAM, which can be classified into independent variables, parameters, and dependent variables, based on the characteristics of each variable (Fayad and Paper, 2015; Kamal et al., 2020). When uncertain factors exist, trust reduces conflicts and risks, promotes decision-making, and maintains a constant relationship (Lăzăroiu et al., 2020). Since live streaming e-commerce is highly dependent on influencers and uncertainties, this study defines user belief in live streaming e-commerce as PT and includes it as a parameter (Ozdemir and Sonmezay, 2020). In this study, independent variables were set as quality factors of e-commerce live streaming users among the various variables verified in ETAM, and the parameters were set as PU, PEU, and PT, while the dependent variable was PI.

### Live Streaming E-Commerce Quality

Live streaming is a format of online business, a marketing and sales method, as well as an upgrade of social e-commerce. While there are many published studies on social e-commerce, scant research on live streaming e-commerce exists. Live streaming eliminates the communication barrier between brands and consumers and focuses on promoting short-term sales by combining brand marketing and performance marketing to enhance corporate marketing efficiency. Since DeLone and McLean (2003) divided the quality characteristics of IS into SQ, IQ, and SEQ, most studies distinguish the quality dimension based on these categories (Shih, 2004). These three qualities were shown to have a key role in customers’ attitudes and acceptance of technologies in Internet shopping (Ahn et al., 2004). The concept and measurement of online shopping quality vary according to the purpose of the study, but most studies refer to quality factors such as IQ, SQ, and SEQ (Hariguna and Berlilana, 2017; Gajewska et al., 2019; Nistah et al., 2019). The quality concepts and measurements of social e-commerce have been used for different purposes, but they have been added or modified based on these three factors (Maia et al., 2018; Wijaya et al., 2019).

Service quality refers to a measure of how much the perceived service coincides with consumer expectations (Pitt et al., 1995), while SEQ refers to providing a consistent service to match consumer expectations Lewis and Booms (1983). The performance measurement of IQ focuses on the value of the information content and characteristics calculated by IQ (Venkatesh et al., 2012). DeLone and McLean (1992) analyzed existing research and showed SQ, in terms of hardware processing information, as being one of the success indicators of an IQ. In this study, based on previous research, the quality factors of live streaming e-commerce were classified into IQ, SQ, and SEQ. The concept of SEQ is defined as the diversity of services provided to live streaming e-commerce users, while IQ pertains to the degree of the system that supports users of live streaming e-commerce to use the service smoothly.

### Perceived Value

Sheth et al. (1991) stated that PV is an abstract belief that is a decision-making standard or a goal of the consumer’s product selection process, which is defined as each consumer being able to purchase the same product with different consumption values or other products with a similar consumption value. Sweeney and Soutar (2001) have proposed that social, emotional, functional, and monetary values are the four sub-dimensions of PV and explained PV as a multidimensional concept that includes consumer decision-making and experience. Lee and Lehto (2013) stated that PV is a relative exchange between what consumers receive through the service and what they provide and that individuals decide to purchase based on value rather than the price.

Perceived value includes the product value, service value, image value, and employee value, as well as investment in time and effort and psychological costs. Customers make purchase decisions based on the PV rather than the price of the products and services. More so, in the live streaming e-commerce environment, consumers make a subjective assessment of product or service value based on individual characteristics or circumstances, since subjective experiences and situations play a large role. Consumers do not require the highest quality of products and services, and they do not always choose the lowest-priced products and services. Live streaming e-commerce provides consumers with products of value for time, effort, and price, based on instant connectivity, time-saving, and perceived convenience.

Perceived value of live streaming e-commerce users is subjectively evaluated by an individual’s situation, given that subjectivity and experience are very important (Tresna et al., 2019). PV refers to the quality or utility value obtained from a purchased product or service. Therefore, this study defined PV of online shopping outlets as the overall service value compared to the overall SEQ.

### Purchase Intention

Purchase intention can be considered as a type of behavior, which is a consumer’s tendency to buy products and services, that is, their willingness to purchase. Beliefs and attitudes toward a specific product are likely to translate into future actions (Engel et al., 1995). Dachyar and Banjarnahor (2017) showed that PI affects the performance of a company and defined consumers as people with positive attitudes toward and beliefs about certain products and who consider purchasing those products (Agmeka et al., 2019).

The PI is a plan of the consumer’s purchase behavior, which leads to purchase behavior through the formation of attitudes and beliefs created on the predictable behavior of the consumer. The PI is a psychological and behavioral tendency, which is formed according to individual attitudes and subjective norms. The PI is formed from the consumer attitude and leads to habitual behaviors, such as repurchase intention and intention to recommend to other shoppers. Companies identify consumers’ PI in advance, predict market conditions, and induce purchases by supplying various experiences and providing information on diverse products and services that reflect consumer demand. Particularly, live streaming e-commerce has data related to many consumers’ PI due to its characteristics and enables consumers to make purchase decisions through various retail channels and promotions according to consumer demand.

Accordingly, this study posits that PI refers to consumers’ subjective willingness and desire intensity to accept and purchase products or services and that it is the most direct factor leading to user purchase behavior. This is mainly reflected in customers’ willingness to accept and use e-commerce of live streaming. In purchase behavior, individuals prioritize the live streaming of goods over the traditional form of e-commerce. Additionally, PI affects the willingness to recommend products or services to friends and family (Dhingra et al., 2020).

### Relationship Between Live Streaming E-Commerce Quality, Perceived Usefulness, and Perceived Ease of Use

Existing research has confirmed the relationship between SEQ, SQ, IQ, PU, and PEU to verify the effects of quality characteristics on innovative products and services (DeLone and McLean, 2003; Sánchez and Hueros, 2010; Venkatesh et al., 2012; Tubaishat, 2018; Sukendro et al., 2020). Scheper et al. (2019) suggested that the quality system of smartphone applications for online to offline services positively impact PU and PEU. In previous studies in various fields, such as online and mobile business, SEQ, SQ, and IQ were proven to positively impact PU and PEU (Rana et al., 2015; Hanjaya et al., 2019).

Therefore, this study hypothesized that the quality of live streaming will positively impact PU and PEU.

*Hypothesis 1*: Live streaming e-commerce quality will positively impact PU.
*Hypothesis 1a*: SQ will positively impact PU.
*Hypothesis 1b*: IQ will positively impact PU.
*Hypothesis 1c*: SEQ will positively impact PU.
*Hypothesis 2*: Live streaming e-commerce quality will positively impact PEU.
*Hypothesis 2a*: SQ will positively impact PEU.
*Hypothesis 2b*: IQ will positively impact PEU.
*Hypothesis 2c*: SEQ will positively impact PEU.

### Relationship Between Live Streaming E-Commerce Quality and Perceived Trust

Gefen (2002) and Eisingerich and Bell (2008) demonstrated through research that SEQ, which is described at the service level, affects consumer trust. Al-Debei et al. (2015) found that the quality of Internet shopping, that is, fashion products, IQ, SQ, and SEQ significantly impact the satisfaction, commitment, and trust of online shoppers. McCole et al. (2010) believed that the most important factor for consumers is the evaluation of specific services in online-based commerce. Trust can affect user judgment and behavior in online environments. McKnight et al. (2017) examined three types of quality and trust in a B2B, found differential impacts of the quality on trusting beliefs, and revealed that IQ had a stronger effect on the trusting beliefs. Sulthana and Vasantha (2021) examined and proved that perceived quality mediated the relationship between social commerce trust and PI.

Based on previous studies, this study hypothesized that the quality of live streaming will positively impact PT.

*Hypothesis 3*: Live streaming e-commerce quality will positively impact PT.
*Hypothesis 3a*: SQ will positively impact PT.
*Hypothesis 3b*: IQ will positively impact PT.
*Hypothesis 3c*: SEQ will positively impact PT.

### Relationship Between Perceived Usefulness, Perceived Ease of Use, Perceived Trust, and Perceived Value

Perceived usefulness increases the value of system use, affects the intention of technology acceptance in TAM (Davis et al., 1989), and is highly useful when new products create new values in terms of function or performance over existing products (Jamal and Sharifuddin, 2015). Zehir et al. (2014) found that usefulness, usability, SQ, and immediate connectivity affect perceived value. In addition, Cheng and Koo (2015) proved that economic, social, and psychological values positively impact usefulness in the relationship between PV and the usefulness of social e-commerce. As a result of analyzing the relationship between perceived hedonic value, PU, and PEU, Mittal et al. (2020) found that perceived hedonic value significantly impacted PU and PEU.

Morgan and Hunt (1994) claimed that shared value had a positive influence on trust, while Jarvenpaa et al. (1999) viewed the trust formation factors of online shopping as PT and the perceived reputation of sellers. In a study of social business by Chinomona et al. (2013), PV was examined in the sub-dimensions of social, informative, financial, emotional, and functional values, and it was verified that PV significantly impacts trust. In accordance with Hernandez-Fernandez and Lewis (2019), consumers’ PV has a positive influence on consumers’ trust in brands and products, which, in turn, plays a key role in maintaining and strengthening the continuous relationship between brands and consumers.

In line with previous studies, this research established the following hypotheses:

*Hypothesis 4*: PU will positively impact PV.
*Hypothesis 5*: PEU will positively impact PV.
*Hypothesis 6*: PT will positively impact PV.

### Relationship Between Perceived Value and Purchase Intention

Zeithaml (1988) pointed out that, from a consumer’s psychological perspective, if they perceive higher benefits for products or services, the sense of value can also improve, and a high PV can increase consumer PI. Dodds (1991) noted that consumer PI was determined by the ratio of the consumer’s perceived gain to the cost paid, and there was a clear positive relationship between PI and PV.

Eggert and Ulaga (2002) recognized that consumers’ PV is the leading factor in promoting consumers’ purchasing behavior. Tam (2004) conducted research on the influence of PV and consumer satisfaction on PI, and consumers’ PV rather than consumers’ satisfaction led consumers to exhibit PI and subsequently execute purchase behavior. Yu et al. (2020) verified that functional and emotional values positively impact continuous use intention through satisfaction.

Based on previous studies, this study hypothesized that PV increases the level of PI.

*Hypothesis 7*: PV will positively impact PI.

## Materials and Methods

We used SPSS 24.0 and AMOS 23.0 to verify the hypotheses. The independent variables were SQ, IQ, and SEQ, the dependent variable was PI, and the mediating variables were PU, PEU, PT, and PV. The research model demonstrates the impacts of the three variables on PI through serial mediation. To verify the hypotheses, we conducted a path analysis.

### Procedures and Respondents

This research aimed to demonstrate the impacts of the quality characteristics of live streaming e-commerce on PI, focusing on ETAM. An online survey was conducted targeting users who had used live streaming service apps (e.g., TikTok, Taobao, and Jingdong) in China. As part of the survey, 300 questionnaires were distributed between January 1, 2021, and March 31, 2021. Of the 272 responses collected, 231 were used for analysis, excluding 41 copies, which contained invalid responses.

The sample characteristics showed 89 men (38.5%) and 142 women (61.5%). Among the participants, 131 (56.7%) were aged less than 30 years, while 85 (36.8%) were aged 31 (5 years. Most of the participants had social networking experience, with 91% having four or more years of experience, while 79% spent 2–3 h per day watching live streaming service apps (e.g., TikTok, Taobao, and Jingdong) in China.

### Measurements

The quality of live streaming e-commerce was categorized into three types: IQ, SQ, and SEQ. Based on the criteria presented by Ahn et al. (2004) and Nistah et al. (2019), which were measured with a total of 12 items to evaluate the quality characteristics of live streaming e-commerce. For PU and PEU, which directly impact users’ attitudes in the TAM, four items were measured based on the studies of Adams et al. (1992) and Venkatesh and Davis (1996), focusing on the effectiveness and convenience of users.

As PT refers to the degree of user belief in live streaming e-commerce, this study included four items with reference to a study by Ozdemir and Sonmezay (2020). PV is regarded as an individual’s subjective evaluation on the selection of a product or a service, which includes aspects, such as cost, time, and effort made by the user (Tresna et al., 2019), and is measured using the four items referred in the study by Shim and Yoon (2020). PI is defined as a positive and friendly behavior intention for live streaming e-commerce and is measured using four items by the instruments of Agmeka et al. (2019). All measurement items in this study were rated on a 5-point Likert scale.

## Results

The findings of confirmatory factor analysis (CFA) showed *X*^2^/df = 1.852 (*p* < 0.001), RMR = 0.039, RMSEA = 0.061, IFI = 0.901, CFI = 0.899, PNFI = 0.695, PGFI = 0.679. Such results are regarded as a good fitness index for the model. The CFA was validated based on these results.

Furthermore, all values of AVE related to variables were higher than 0.5 (SQ = 0.556, IQ = 0.624, SEQ = 0.650, PEU = 0.565, PU = 0.593, PT = 0.629, PV = 0.572, PI = 0.585). The results of constricting reliability of all variables were higher than 0.7 (SQ = 0.871, IQ = 0.934, SEQ = 0.910, PEU = 0.826, PU = 0.813, PT = 0.887, PV = 0.896, PI = 0.873). Based on these results, the concentration validity was shown to be significant.

The results of Cronbach’s α for reliability analysis showed that all variables were higher than 0.70 (SQ = 0.822, IQ = 0.866, SEQ = 0.863, PEU = 0.705, PU = 0.768, PT = 0.880, PV = 0.803, PI = 0.846). Therefore, Cronbach’s α for the reliability analysis was significant.

In Table 1, the results of the correlation analysis revealed that SQ (*r* = 0.209, *p* < 0.01), IQ (*r* = 0.133, *p* < 0.05), and SEQ (*r* = 0.236, *p* < 0.001) were positively related to PU. In addition, IQ (*r* = 0.165, *p* < 0.05) and SEQ (*r* = 0.145, *p* < 0.05) were positively associated with PEU. However, SEQ (*r* = 0.054, *p* > 0.05) and PEU were insignificant. Moreover, SQ (*r* = 0.205, *p* < 0.01), IQ (*r* = 0.151, *p* < 0.05), and SEQ (*r* = 0.137, *p* < 0.001) were positively related to PV.

**TABLE 1:** Results of correlation analysis.

	Mean	SD	1	2	3	4	5	6	7	8
1	3.069	0.658	–							
2	3.688	0.533	0.179**	–						
3	3.737	0.690	0.150*	0.238***	–					
4	3.746	0.786	0.209**	0.133*	0.236***	–				
5	3.804	0.635	0.054	0.165*	0.145*	0.231***	–			
6	2.956	0.804	0.205**	0.151*	0.137*	0.006	0.069	–		
7	3.301	0.575	0.180**	0.204**	0.015	0.160*	0.067	0.231***	–	
8	3.573	0.761	0.100	0.157*	−0.078	−0.020	0.084	0.027	0.203**	–

*N = 231; 1, SQ; 2, IQ; 3, SEQ; 4, PU; 5, EU; 6, PT; 7, PV; 8, PI; SQ, system quality; IQ, information quality; SEQ, service quality; PU, perceived usefulness; PEU, perceived ease of use; PT, perceived trust; PV, perceived value; PI, purchase intention.*

***p < 0.001, **p < 0.01, *p < 0.05.

PU (*r* = 0.160, *p* < 0.05) and PT (*r* = 0.231, *p* < 0.001) were positively related to PV. However, the correlation analysis between PEU (*r* = 0.067, *p* > 0.05) and PV was not significant. Finally, PV (*r* = 0.203, *p* < 0.01) was positively related to PI.

Path analysis 1 was used to test the hypotheses. The results showed that the system positively impacts PU (estimate = 0.270, *p* < 0.01) and PT (estimate = 0.218, *p* < 0.01). However, the influence of SQ on PEU (estimate = 0.014, *p* > 0.10) was not significant. Information positively impacts PEU (estimate = 0.140, *p* < 0.01) and PT (estimate = 0.169, *p* < 0.01). However, the influence of SQ on PU (estimate = 0.118, *p* > 0.10) was not significant. Service had a positive effect on PU (estimate = 0.386, *p* < 0.01) and PEU (estimate = 0.133, *p* < 0.10). However, the effect of service on PT (estimate = 0.131, *p* > 0.10) was not significant. PU (estimate = 0.157, *p* < 0.01) and PT (estimate = 0.202, *p* < 0.01) had a positive effect on PV. However, the effect of PEU on PV (estimate = 0.039, *p* > 0.10) was not significant. Finally, PV positively impacts PI (estimate = 0.260, *p* < 0.001).

Table 2 is the path (SQ → PEU → PV → PI) analysis of the effect of SQ on PI. First, the effect of SQ on PEU was verified. The result showed that the effect of SQ on PEU is not significant (estimate = 0.051, *p* > 0.10). It showed the value of the estimate is 0.051 and the p-value is higher than 0.10. Second, the effect of PEU on PV was verified. The result showed that the effect of PEU on PV is also insignificant (estimate = 0.121, *p* > 0.10). Third, the effect of PV on PI was verified. The result showed that the effect of PV on PI is significant (estimate = 0.265, *p* < 0.001). Finally, the indirect effect was verified. The result showed that the indirect effect is 0.002. Bootstrapping showed that the lower and upper bounds are 0.000 and 0.000, respectively. The goodness of model fit showed *X*^2^/df = 1.173 (*p* > 0.05), RMR = 0.042, RMSEA = 0.027, IFI = 0.988, CFI = 0.987, TLI = 0.985, PNFI = 0.763, and PGFI = 0.687. According to these results, although the goodness of model fit is significant, the effect of SQ on PEU is not significant. Furthermore, the effect of PEU on PV is also insignificant. Thus, the path analysis is insignificant and hypothesis 2 is rejected.

**TABLE 2:** Results of path analysis 2.

Path	Estimate	Standard error	Critical ratio	*p*
The effect of SQ on PEU	0.051	0.063	0.813	–
The effect of PEU on PV	0.121	0.105	1.152	–
The effect of PV on PI	0.265	0.079	3.372	***

**Path**	**Indirect effect**		**Boot LLCI**	**Boot ULCI**

SQ→PEU→PV→PI	0.002		0.000	0.000

*N = 231; SQ, system quality; PEU, perceived ease of use; PV, perceived value; PI, purchase intention.*

***p < 0.001, **p < 0.01, *p < 0.05.

Table 3 is the path (IQ → PU → PV → PI) analysis of the effect of IQ on the PI. First, the effect of IQ on PU was verified. The result showed that the effect of IQ on PU is significant (estimate = 0.267, *p* < 0.05). It showed that the value of the estimate is 0.267 and the value of *p* is lower than 0.05. Second, the effect of PU on PV was verified. The result showed that the effect of PU on PV is also significant (estimate = 0.173, *p* < 0.01). Third, the effect of PV on PI was verified. The result showed that the effect of PV on PI is significant (estimate = 0.262, *p* < 0.001). Finally, the indirect effect was verified. The result showed that the indirect effect is 0.010. Bootstrapping showed that the lower and upper bounds are 0.001 and 0.039, respectively. The goodness of model fit showed *X*^2^/df = 1.462 (*p* < 0.05), RMR = 0.045, RMSEA = 0.045, IFI = 0.972, CFI = 0.971, TLI = 0.965, PNFI = 0.758, and PGFI = 0.675. According to these results, the goodness of model fit is significant, the effect of IQ on PU is significant, the effect of PU on PV is also significant. Furthermore, the effect of PV on PI is significant and the indirect effect is also significant. Thus, the path analysis is significant and hypothesis 3 is supported.

**TABLE 3:** Results of path analysis 3.

Path	Estimate	Standard error	Critical ratio	*p*
The effect of IQ on PU	0.267	0.119	2.241	*
The effect of PU on PV	0.173	0.062	2.813	**
The effect of PV on PI	0.262	0.079	3.339	***

**Path**	**Indirect effect**		**Boot LLCI**	**Boot ULCI**

IQ→PU→PV→PI	0.010		0.001	0.039

*N = 231; IQ, information quality; PU, perceived usefulness; PV, perceived value; PI, purchase intention.*

***p < 0.001, **p < 0.01, *p < 0.05.

Table 4 is the path (IQ → PEU → PV → PI) analysis of the effect of IQ on PI. First, the effect of IQ on PEU was verified. The result showed that the effect of IQ on PEU is significant (estimate = 0.175, *p* < 0.05). It showed that the value of the estimate is 0.175 and the p-value is lower than 0.05. Second, the effect of PEU on PV was verified. The result showed that the effect of PEU on PV is insignificant (estimate = 0.132, *p* > 0.05). Third, the effect of PV on PI was verified. The result showed that the effect of PV on PI is significant (estimate = 0.265, *p* < 0.001). Finally, the indirect effect was verified. The result showed that the indirect effect is 0.005. Bootstrapping showed that the lower and upper bounds are −0.001 and 0.029, respectively. The goodness of model fit showed *X*^2^/df = 1.285 (*p* < 0.05), RMR = 0.041, RMSEA = 0.035, IFI = 0.982, CFI = 0.981, TLI = 0.977, PNFI = 0.764, and PGFI = 0.681. According to these results, although the goodness of model fit is significant and the effect of IQ on PEU is also significant, the effect of PEU on PV is not significant and the indirect effect is also insignificant. Thus, the path analysis is insignificant and hypothesis 4 is rejected.

**TABLE 4:** Results of path analysis 4.

Path	Estimate	Standard error	Critical ratio	*p*
The effect of IQ on PEU	0.175	0.076	2.307	*
The effect of PEU on PV	0.132	0.106	1.245	–
The effect of PV on PI	0.265	0.078	3.374	***

**Path**	**Indirect effect**		**Boot LLCI**	**Boot ULCI**

IQ→PEU→PV→PI	0.005		−0.001	0.029

*N = 231; IQ, information quality; PEU, perceived ease of use; PV, perceived value; PI, purchase intention.*

***p < 0.001, **p < 0.01, *p < 0.05.

Table 5 is the path (IQ → PT → PV → PI) analysis of the effect of IQ on the PI. First, the effect of IQ on PT was verified. The result showed that the effect of IQ on PT is significant (estimate = 0.255, *p* < 0.05). It showed that the value of the estimate is 0.255 and the *p*-value is lower than 0.05. Second, the effect of PT on PV was verified. The result showed that the effect of PT on PV is significant (estimate = 0.209, *p* < 0.01). Third, the effect of PV on PI was verified. The result showed that the effect of PV on PI is significant (estimate = 0.262, *p* < 0.001). Finally, the indirect effect was verified. The result showed that the indirect effect is 0.012. Bootstrapping showed that the lower and upper bounds are 0.000 and 0.042, respectively. The goodness of model fit showed *X*^2^/df = 2.988 (*p* < 0.05), RMR = 0.046, RMSEA = 0.093, IFI = 0.894, CFI = 0.893, TLI = 0.872, PNFI = 0.714, and PGFI = 0.649. According to these results, although the effect of IQ on PT and the effect of PT on PV are significant, the goodness of model fit and indirect effect is insignificant. Thus, the path analysis is insignificant and hypothesis 5 is rejected.

**TABLE 5:** Results of path analysis 5.

Path	Estimate	Standard error	Critical ratio	*p*
The effect of IQ on PT	0.255	0.102	2.516	*
The effect of PT on PV	0.209	0.064	3.273	**
The effect of PV on PI	0.262	0.078	3.359	***

**Path**	**Indirect effect**		**Boot LLCI**	**Boot ULCI**

IQ→PT→PV→PI	0.012		0.000	0.042

*N = 231; IQ, information quality; PT, perceived trust; PV, perceived value; PI, purchase intention.*

***p < 0.001, **p < 0.01, *p < 0.05.

Table 6 is the path (SEQ → PEU → PV → PI) analysis of the effect of SEQ on the PI. First, the effect of SEQ on PEU was verified. The result showed that the effect of SEQ on PEU is significant (estimate = 0.162, *p* < 0.05). It showed that the value of the estimate is 0.162 and the *p*-value is lower than 0.05. Second, the effect of PEU on PV was verified. The result showed that the effect of PEU on PV is insignificant (estimate = 0.118, *p* > 0.10). Third, the effect of PV on PI was verified. The result showed that the effect of PV on PI is significant (estimate = 0.265, *p* < 0.001). Finally, the indirect effect was verified. The result showed the indirect effect is 0.004. Bootstrapping showed that the lower and upper bounds are −0.002 and 0.022, respectively. The goodness of model fit showed *X*^2^/df = 1.213 (p > 0.05), RMR = 0.036, RMSEA = 0.030, IFI = 0.986, CFI = 0.986, TLI = 0.983, PNFI = 0.767, PGFI = 0.684. According to these results, although the goodness of model fit is significant and the effect of SEQ on PEU is also significant, the effect of PEU on PV is not significant and the indirect effect is also insignificant. Thus, the path analysis is insignificant and hypothesis 6 is rejected.

**TABLE 6:** Results of path analysis 6.

Path	Estimate	Standard error	Critical ratio	*p*
The effect of SEQ on PEU	0.162	0.074	2.185	*
The effect of PEU on PV	0.118	0.106	1.111	–
The effect of PV on PT	0.265	0.079	3.371	***

**Path**	**Indirect effect**		**Boot LLCI**	**Boot ULCI**

SEQ→PEU→PV→PI	0.004		−0.002	0.022

*N = 231; SEQ, service quality; PEU, perceived ease of use; PV, perceived value; PI, purchase intention.*

***p < 0.001, **p < 0.01, *p < 0.05.

Table 7 is the path (SEQ → PT → PV → PI) analysis of the effect of SEQ on the PI. First, the effect of SEQ on PT was verified. The result showed that the effect of SEQ on PT is significant (estimate = 0.213, *p* < 0.05). It showed that the value of the estimate is 0.213 and the *p*-value is lower than 0.05. Second, the effect of PT on PV was verified. The result showed that the effect of PT on PV is significant (estimate = 0.207, *p* < 0.01). Third, the effect of PV on PI was verified. The result showed that the effect of PV on PI is significant (estimate = 0.262, *p* < 0.001). Finally, the indirect effect was verified. The result showed that the indirect effect is 0.010. Bootstrapping showed that the lower and upper bounds are 0.000 and 0.040, respectively. The goodness of model fit showed *X*^2^/df = 1.659 (*p* < 0.05), RMR = 0.037, RMSEA = 0.054, IFI = 0.962, CFI = 0.962, TLI = 0.955, PNFI = 0.766, and PGFI = 0.680. According to these results, although the goodness of model fit is significant, the effect of SEQ on PT and the effect of PV on PI are also significant, but the indirect effect (SEQ → PT → PV) is insignificant (lower bounds = −0.001, upper bounds = 0.113). Thus, the path analysis is insignificant and hypothesis 7 is rejected.

**TABLE 7:** Results of path analysis 7.

Direct effect	Estimate	Standard error	Critical ratio	*p*
The effect of SEQ on PT	0.213	0.099	2.143	*
The effect of PT on PV	0.207	0.064	3.228	**
The effect of PV on PI	0.262	0.078	3.357	***

**Path**	**Indirect effect**		**Boot LLCI**	**Boot ULCI**

SEQ→PT→PV→PI	0.010		0.000	0.040

*N = 231; SEQ, service quality; PT, perceived trust; PV, perceived value; PI, purchase intention.*

***p < 0.001, **p < 0.01, *p < 0.05.

Table 8 is path analysis 9 (SQ → PU → PV → PI), which showed that the system positively impacts PU (estimate = 0.341, *p* < 0.001), and PU positively impacts PV (estimate = 0.175, *p* < 0.01). Furthermore, PV positively impacts PI (estimate = 0.262, *p* < 0.001). The indirect effect is 0.016. Bootstrapping showed that the lower and upper bounds are 0.004 and 0.048, respectively. The goodness of model fit showed *X*^2^/df = 1.276 (*p* < 0.05), RMR = 0.042, RMSEA = 0.035, IFI = 0.981, CFI = 0.981, TLI = 0.977, PNFI = 0.762, and PGFI = 0.683. According to these results, path analysis 8 is significant.

**TABLE 8:** Results of path analysis 8.

Direct effect	Estimate	Standard error	Critical ratio	*p*
The effect of SQ on PU	0.341	0.100	3.393	***
The effect of PU on PV	0.175	0.061	2.853	**
The effect of PV on PI	0.262	0.079	3.337	***

**Path**	**Indirect effect**		**Boot LLCI**	**Boot ULCI**

SQ→PU→PV→PI	0.016		0.004	0.048

*N = 231; SQ, system quality; PU, perceived usefulness; PV, perceived value; PI, purchase intention.*

***p < 0.001, **p < 0.01, *p < 0.05.

Table 9 is path analysis 10 (SQ → PT → PV → PI), which showed that the system positively impacts PT (estimate = 0.266, *p* < 0.01). PT also positively impacts PV (estimate = 0.211, *p* < 0.01). Furthermore, PV positively impacts PI (estimate = 0.262, *p* < 0.001). The indirect effect is 0.015. Bootstrapping showed that the lower and upper bounds are 0.003 and 0.054, respectively. The goodness of model fit showed X2/df = 1.793 (*p* < 0.001), RMR = 0.041, RMSEA = 0.059, IFI = 0.952, CFI = 0.951, TLI = 0.942, PNFI = 0.755, and PGFI = 0.680. According to these results, path analysis 9 is significant.

**TABLE 9:** Results of path analysis 9.

Direct effect	Estimate	Standard error	Critical ratio	*p*
The effect of SQ on PT	0.266	0.084	3.172	**
The effect of PT on PV	0.211	0.064	3.275	**
The effect of PV on PI	0.262	0.078	3.358	***

**Path**	**Indirect effect**		**Boot LLCI**	**Boot ULCI**

SQ→PT→PV→PI	0.015		0.003	0.054

*N = 231; SQ, system quality; PT, perceived trust; PV, perceived value; PI, purchase intention.*

***p < 0.001, **p < 0.01, *p < 0.05.

Table 10 is path analysis 11 (SEQ → PU → PV →PI), which showed that SEQ positively impacts PU (estimate = 0.461, *p* < 0.001). PU positively impacts PV (estimate = 0.159, *p* < 0.01). Furthermore, PV positively impacts PI (estimate = 0.262, *p* < 0.001). The indirect effect is 0.016. Bootstrapping showed that the lower and upper bounds are 0.005 and 0.046, respectively. The goodness of model fit showed *X*^2^/df = 1.202 (*p* > 0.05), RMR = 0.040, RMSEA = 0.030, IFI = 0.988, CFI = 0.987, TLI = 0.985, PNFI = 0.771, and PGFI = 0.685. According to these results, path analysis 10 is significant.

**TABLE 10:** Results of path analysis 10.

Direct effect	Estimate	Standard error	Critical ratio	*p*
The effect of SEQ on PU	0.461	0.122	3.792	***
The effect of PU on PV	0.159	0.059	2.686	**
The effect of PV on PI	0.262	0.079	3.337	***

**Path**	**Indirect effect**		**Boot LLCI**	**Boot ULCI**

SEQ→PU→PV→PI	0.016		0.005	0.046

*N = 231; SEQ, service quality; PU, perceived usefulness; PV, perceived value; PI, purchase intention.*

***p < 0.001, **p < 0.01, *p < 0.05.

## Discussion

### Contributions and Implications

This study examined the influence of live streaming e-commerce’s quality characteristics, such as SQ, IQ, and SEQ, on consumer PI by applying the ETAM. Additionally, this study analyzes the differences in the effect of live streaming e-commerce’s quality characteristics on consumer PI based on the paths of PU, PEU, PT, and PV.

Based on the results of empirical analysis, the findings of this study are shown as follows: First, SQ, which constitutes the quality characteristics of live streaming e-commerce, significantly impacts PU and PT. However, SQ has not been found to have a significant effect on PEU, suggesting that the level of a system that supports live streaming e-commerce users to use services smoothly is not easy.

Second, IQ, which constitutes the quality characteristics of live streaming e-commerce, has a significant effect on PEU and PT. However, quality characteristics have an insignificant effect on PU, suggesting that information obtained from live streaming e-commerce is not useful. Therefore, it is necessary to find ways for users to find various types of information about products provided by live streaming e-commerce easily and efficiently.

Third, the SEQ, which constitutes the quality characteristics of live streaming e-commerce significantly, impacts PU and PEU. However, SEQ has an insignificant impact on PT, which shows the limitation of trust in processing inquiries or inconveniences that occur during e-commerce of live streaming. Therefore, it should be emphasized to determine users’ reliability through the management of SEQ aspects, such as by providing accurate information about products and friendly problem-solving.

Fourth, according to the path analysis results, the SQ of live streaming e-commerce significantly impacts the value of perception through PU and PT and finally has a positive effect on the PI. Influencers act as shopping guides for consumers using live streaming e-commerce to shop, explaining related information of products to users, thereby forming a mutual trust. Owing to the explosive popularity of live streaming e-commerce in China, many people are engaged in the business, and users are gradually losing trust due to fake information, such as false publicity. Therefore, to develop sustainable live streaming e-commerce, it is important to establish a system that can provide consumers trust, focusing on live streaming platform companies, companies, and influencers.

Fifth, the SEQ of live streaming e-commerce significantly impacts PV through PU and consequently has a positive impact on the PI. According to this, live streaming e-commerce explains the information, as well as advantages and disadvantages of the product in detail to solve problems that cannot be directly experienced and shows it in the form of a live video even in the latter period of use. This approach helps dispense information about the product more accurately than photographs or phrases, and thus, it makes it easier to understand how to use products and to avoid losses on account of wrong purchases. In addition, live streaming e-commerce is conducted in real-time and user queries can be answered instantly, providing two-way communication. Users’ participation and communication can promote consumers’ purchase behavior and make them experience the pleasure of shopping.

Finally, the principle implications are summarized as follows: The COVID-19 pandemic has affected various aspects of retail. China is at the forefront of innovation in online retail worldwide. In particular, live-streaming e-commerce is gaining traction as a current mainstream consumer form, suggesting that its popularity meets market demand. In addition, this study explores which factors influence consumers’ decisions related to the use of live streaming e-commerce and emphasizes the importance of SQ and SEQ for increasing financial institutions’ and enterprises’ performances. These two factors can increase PI.

### Limitations and Future Research

This study has limitations in various aspects. We have also provided the directions for future study. Both limitations and the direction of future study are presented as follows: First, the sample used in the analysis was targeted at Chinese consumers, and there were more female than male participants, leading to a generalization limitation. Therefore, future studies should focus on consumers in many countries. Second, while this study added the variables of PT and PV, it will be necessary to add various external and moderate variables that affect e-commerce of live streaming in the future.

### Conclusion

This study verified the role of live streaming e-commerce quality, such as SQ, IQ, and SEQ, on consumer PI in China by applying ETAM. Additionally, it was verified that there were differences in the impact of live streaming e-commerce quality on PI, depending on the paths of PU, PEU, PT, and PV. This study confirmed that SQ of live streaming e-commerce affected the PV through PU and PT significantly and ultimately influenced consumer PI. This suggests that consumers value SQ, such as stable operating and transaction processing systems and an easy access environment when using live streaming e-commerce. Moreover, the path in which SEQ affects PU, which in turn affects PV, and ultimately PI, was found. It could be inferred that polite and prompt resolution of issues faced by consumers and delivery of useful information can strengthen consumer PI. Notwithstanding some limitations of this study, it presents factors that can strengthen consumer PI using live streaming e-commerce and it is also expected to provide data for the strategic development of various industries.

## Data Availability Statement

The original contributions presented in this study are included in the article/supplementary material, further inquiries can be directed to the corresponding author/s.

## Ethics Statement

The studies involving human participants were reviewed and approved by the Gachon University. The patients/participants provided their written informed consent to participate in this study. Written informed consent was obtained from the individual(s) for the publication of any potentially identifiable images or data included in this article.

## Author Contributions

CQ performed the data collection and analysis. SJ contributed to drafting, review, and editing. Both authors contributed to the study’s conception and design.

## Conflict of Interest

The authors declare that the research was conducted in the absence of any commercial or financial relationships that could be construed as a potential conflict of interest.

## Publisher’s Note

All claims expressed in this article are solely those of the authors and do not necessarily represent those of their affiliated organizations, or those of the publisher, the editors and the reviewers. Any product that may be evaluated in this article, or claim that may be made by its manufacturer, is not guaranteed or endorsed by the publisher.
